# Differential Training Facilitates Early Consolidation in Motor Learning

**DOI:** 10.3389/fnbeh.2016.00199

**Published:** 2016-10-21

**Authors:** Diana Henz, Wolfgang I. Schöllhorn

**Affiliations:** Institute of Sport Science, Training and Movement Science, University of MainzMainz, Germany

**Keywords:** motor learning, variable practice, differential learning, repetitive learning, EEG, badminton

## Abstract

Current research demonstrates increased learning rates in differential learning (DL) compared to repetitive training. To date, little is known on the underlying neurophysiological processes in DL that contribute to superior performance over repetitive practice. In the present study, we measured electroencephalographic (EEG) brain activation patterns after DL and repetitive badminton serve training. Twenty-four semi-professional badminton players performed badminton serves in a DL and repetitive training schedule in a within-subjects design. EEG activity was recorded from 19 electrodes according to the 10–20 system before and immediately after each 20-min exercise. Increased theta activity was obtained in contralateral parieto-occipital regions after DL. Further, increased posterior alpha activity was obtained in DL compared to repetitive training. Results indicate different underlying neuronal processes in DL and repetitive training with a higher involvement of parieto-occipital areas in DL. We argue that DL facilitates early consolidation in motor learning indicated by post-training increases in theta and alpha activity. Further, brain activation patterns indicate somatosensory working memory processes where attentional resources are allocated in processing of somatosensory information in DL. Reinforcing a somatosensory memory trace might explain increased motor learning rates in DL. Finally, this memory trace is more stable against interference from internal and external disturbances that afford executively controlled processing such as attentional processes.

## Introduction

Understanding how to make motor learning more efficient and effective is an important goal in behavioral neuroscience. The application of variable practice has achieved acceptance as being beneficial for motor learning processes. Numerous studies have demonstrated enhanced motor learning performance in variable practice over repetitive learning schedules (for an overview see Beckmann, 2013). An important research question in literature raises the discussion on type, schedule and amount of variation to be applied in motor learning settings. Different training schedules and degrees of variations have been suggested for varied acquisition in the context of motor learning. First, models of variation were included in methodical rows of exercises whereby the motor task is approached by exercises that become subsequently more similar to the goal task (Gaulhofer and Streicher, 1924). Based on the theoretical assumption that similar movements can be condensed into classes of movements that can be modeled by means of invariants and variable parameters (Schmidt, 1975), varied training with the same invariants and several variable parameters was suggested to result in more stable generalized motor programs, and therefore with superior performance in retention and transfer of the learned motor task (Moxley, 1979).

A different theoretical perspective on variable practice was introduced by the system dynamic approach (Haken, 1970; Glansdorff and Prigogine, 1971). In contrast to previous learning approaches, the differentiation between errors and variations was replaced by the more neutral term of *fluctuations* derived from physics. One of the main characteristics of dissipative systems that have been investigated by the system dynamic approach is that living systems show fluctuations continuously and an increase of fluctuations before a phase transition. This observed increase of fluctuations constitutes the basis for a self-organizing process in a way that the system is exploring different modes during the increased fluctuations in order to find a new and more effective mode. Within the differential learning (DL) approach, fluctuations during the motor learning process are a fundamental basis for motor learning. In contrast, in repetition based motor learning movements are performed with a large number of repetitions without voluntary variations until a predefined ideal state of movement is reached. Based on these theoretical assumptions, the DL approach has been applied in the context of motor learning and extensively investigated by Schöllhorn and collaborates (Schöllhorn, 1999, 2000; Schöllhorn et al., 2006, 2009, 2010; Frank et al., 2008).

During a goal kicking experiment, a group of soccer players were trained according to the DL approach with no movement repetition while another group trained with repetitions (Schöllhorn et al., 2009). The DL group trained the kicking movement, e.g., with stiff knees or stiff hip, slack foot and circling arms, hopping run up and arbitrary combinations of different tasks on the kicking and standing leg as well as in the trunk, head and arm positions. In difference, the repetitional training group followed exact prescriptions that were based on a movement prototype, and ample repetitions. After 4 weeks of intervention and the same amount of kicks in both groups, the DL group showed a significantly higher progress in kicking precision in comparison to the repetitional group.

Increased acquisition rates in DL compared to repetitive training were shown for handball (Wagner and Müller, 2008), basketball (Lattwein et al., 2014), volleyball (Römer et al., 2009), track and field (Beckmann and Schöllhorn, 2006; Beckmann and Gotzes, 2009), ice-skating (Savelsbergh et al., 2010), hockey (Beckmann et al., 2010) and even for two football movements in parallel (Schöllhorn et al., 2012). Most intriguingly, DL not only leads to increased acquisition rates but also to increased learning rates (Beckmann and Schöllhorn, 2006; Savelsbergh et al., 2010). After 4 weeks of scheduled creative movement variations that are from a traditional point of view considered as movement errors and accordingly have to be avoided in common motor learning schedules, the DL group displayed further improvements up to 4 weeks after the intervention ended. In contrast, the performances of the repetitional training group dropped back to the original level already after 2 weeks post intervention (Beckmann and Schöllhorn, 2006). Further evidence is given on the effect of DL on the postural sway (James, 2014). In a recent clinical trial, faster recovery of arm function in stroke patients after DL compared to repetitive training was shown (Repšaitė et al., 2015).

Most studies conducted on effects of learning in general and motor learning, investigate event-related brain potentials. To date, little is known on the effects of motor learning on spontaneous electroencephalographic (EEG) brain oscillations. Studies in humans have shown that learning leaves local traces that can be detected immediately after the performance (Tanaka et al., 2011; Buschkuehl et al., 2012; Crupi et al., 2013). The notion that post-performance traces are local and task-specific, has been confirmed by recent studies. Theta increases in parieto-occipital areas after a driving video game (Hung et al., 2013), or after adaptation to a rotated display (Ghilardi et al., 2000; Krakauer et al., 2000; Huber et al., 2004) were demonstrated. Alpha power changes in the resting-state EEG were found in regions that showed EEG changes during the task (Landsness et al., 2011; Perfetti et al., 2011). Moisello et al. (2013) demonstrated theta increases in frontal and posterior regions with alpha increases in the spontaneous EEG following a sequence-learning task. These post-task changes may represent a trace of learning and a hallmark of use-dependent plasticity.

Several studies have investigated the underlying neurophysiological processes that lead to better performance in interleaved over repetitive practice. Neurophysiological studies show differences in cortical activation in interleaved compared to repetitive practice with increased activity in sensorimotor and premotor regions (Cross et al., 2007), in the premotor-parietal network and sensorimotor and subcortical regions (Wymbs and Grafton, 2009). These areas are associated with motor preparation, sequencing and response selection. Further, several studies identified activation of fronto-parietal networks in CI (Serrien, 2009; Lin et al., 2011, 2012, 2013). More specifically, activation in the dorsal premotor (PM) and the dorsolateral prefrontal (DLPFC) cortices changed in interleaved practice. Further, increased inter-regional functional connectivity in CI compared to repetitive training for both PM-seeded and DLPFC-seeded connectivity was demonstrated. The observed patterns of brain activation indicated the formation of enhanced memory traces and efficient long-term retrieval in interleaved practice. In an EEG study, differences in brain activation in interleaved and repetitive practice were demonstrated (Tanaka et al., 2005).

Despite the systematically demonstrated effects of DL on motor learning, little is currently known about the neural basis of how DL leads to better performance than repetitive training. To our knowledge, this is the first study that compares effects of DL and repetitive training on EEG brain activity. First evidence for neurophysiological post-training effects comes from three pilot studies that examined EEG brain activity after DL, compared to repetitive training. Increased frontal and central EEG theta activity with central posterior alpha activity was obtained after DL in badminton serve training (Henz et al., 2013, 2015) and in soccer goal shooting training (Henz et al., 2014).

The present study was designed to measure acute effects of DL and repetitive badminton serve training on spontaneous EEG brain activation patterns in an experimental design. We predicted that different brain activation patterns would be demonstrated in DL compared to repetitive training. We argue that according to the results found in prior studies, increased somatosensory EEG theta activity and posterior and central alpha activity are increased due to the specific characteristics of DL that stimulates the motor and somatosensory areas extensively, and due to its high affordances on motor control.

## Materials and Methods

### Participants

Twenty-four semi-professional badminton players (mean age: 25.3 years; age range: 18–34; 16 males, 8 females) volunteered in this study. Informed consent was obtained from all participants and all procedures were conducted in accordance with the ethical standards of the Helsinki Declaration of the World Medical Association Assembly. Participants had at least regular badminton training experience of 1 year. All participants were healthy, and had no current diseases or a history of neurological impairments or intake of medication that may have affected EEG recordings. All subjects were naïve as to the purpose of the current study. All subjects gave written informed consent. The experimental procedures were approved by the local ethics committee at the Johannes Gutenberg University of Mainz, Germany. All experimental procedures were carried out in accordance with the Declaration of Helsinki.

### EEG Recording Details

The EEG was recorded by using the Micromed Brainquick amplifier (SD-LTM-32) and Micromed Brainspy software (Micromed, Venice, Italy). Recordings were made from Fp1, Fp2, F3, F7, Fz, F4, F8, C3, Cz, C4, T3, T4, P3, P7, Pz, P4, P8, O1, O2 placed according to the Int. 10–20 system with reference to the nose. All electrode impedances were kept at 5 kΩ or below. The EEG signals were continuously recorded and digitized at a sampling rate of 256 Hz. The EEG signal was amplified with a time constant of 0.3 s with a second order high-pass filter at 0.5 Hz and a low-pass filter at 120 Hz (frequency range: 0.5–120 Hz). Electrooculography (EOG) was monitored placed at the medial upper and lateral orbital rim of the right eye (time constant: 0.3 s; high pass filter: 0.1 Hz; low pass filter: 120 Hz; frequency range: 0.5–120 Hz). Two electrodes placed on the neck and on the shoulder recorded muscle activity. Heart rate (Polar S810i, Polar Electro, Buettelborn, Germany) was assessed continuously to control exercise intensity.

### Experimental Procedure

Before the experiment, the tasks were explained. Participants were shown the appropriate grip for each serve, where and how to stand as well as how to move. Performance in badminton serves was assessed prior to the single training intervention. Subjects performed 60 badminton serves towards a target located at the left service court of the playing field at a distance of 8.40 m from the service line. The subject stood in the right service court and performed all serves from the service line of the right court. Initial performance was measured on the day before the training intervention. On the consecutive day, the training intervention with EEG measurement was performed. At each measurement time point, participants began with a resting condition followed by the recording of spontaneous EEG of the subject for 4 min with eyes-open. Then, they were required to perform a 20-min training session, followed by a 4 min rest with eyes-open. The experiment contained two tasks for each subject. Participants performed a differential and a repetitive badminton serve training in a within-subjects design. Badminton serves were performed with the right hand towards eight segments of the service court. In repetitive training, badminton serves were performed without movement variations. In DL, badminton serves were performed in three blocks with variation of one, two and three parameters at the same time. In the DL condition, none of the practice trials was repeated for more than three times. The number of badminton serves was defined for each experimental condition. Sixty trials were performed in repetitive training and 60 trials in DL. The experimental conditions were tested on two consecutive days. Experimental conditions were randomized. EEG data were obtained during the three resting conditions: (1) pre-training rest; (2) post-DL rest; and (3) post-repetitive training rest, which were then taken for subsequent analyses.

### EEG Analysis

The spontaneous EEG was recorded for 4 min with eyes-open before and after each experimental condition. The EEG and EOG signals were visually scored and portions of the data that contained aberrant eye movements and muscle movements of artifacts were removed. The EEG was analyzed and Fast Fourier Transforms were used to obtain the mean power amplitudes in theta (4–7.5 Hz), alpha (8–13 Hz), beta (14–30 Hz) and gamma (31–40 Hz) bands.

### Statistical Analyses

Means and standard deviations of hit ratios of badminton serves in the initial test were calculated. The reliability of the measurement scale was obtained by calculation of Cronbach’s alpha as a measure of internal consistency. Hit ratios of each training condition were subjected to a repeated-measure analysis of variance (ANOVA) including the within-subjects factor training (repetitive training, DL, control baseline rest), followed by a Bonferroni-corrected *post hoc* test for further comparisons. A statistical comparison of power data of the theta, alpha, beta and gamma bands was calculated by repeated-measure ANOVA including the within-subject factors as experimental condition (baseline rest, repetitive training, DL) and location (Frontal, Central, Temporal, Parietal, Occipital), followed by Bonferroni-corrected *post hoc* tests for further comparisons. Effects were considered to be statistically significant when the *p*-values were less than 0.05.

## Results

### Performance Errors

**Figure 1:**
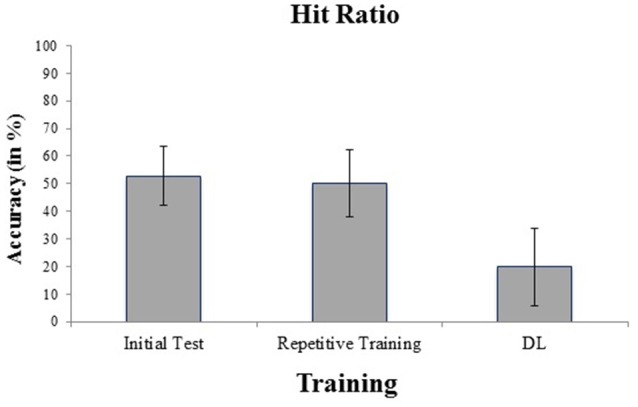
**Hit ratios in the initial test, differential learning (DL) and repetitive badminton serve training.** Decreased hit ratios are reached in DL compared to repetitive training, and the initial test.

Figure 1 show means and standard deviations of hit ratios in badminton serves for the initial test, and for each training condition. Cronbach’s alpha for the initial test revealed a high internal consistency, *α* = 0.89. The ANOVA of hit ratios in the training intervention revealed a highly significant effect for training, *F*_(2,46)_ = 25.62, *p* = 0.0014, $\eta_{p}^{2}$ = 0.31.

### Statistical Description: Spontaneous EEG

**Figure 2:**
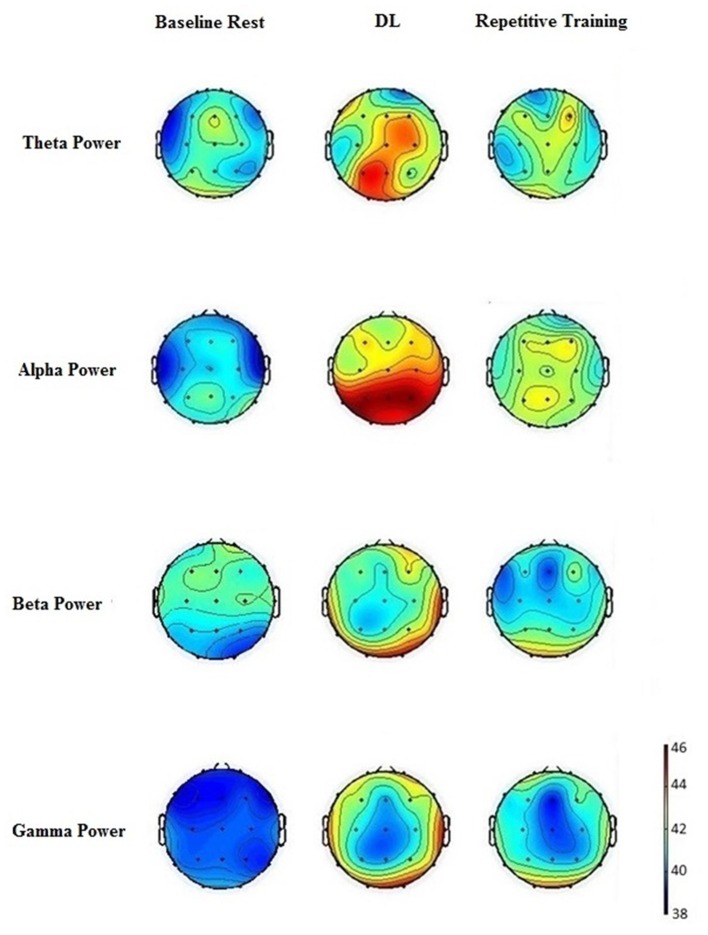
**Spontaneous electroencephalographic (EEG) brain activity at baseline rest, after DL and repetitive badminton serve training.** Theta activity is increased in contralateral parieto-occipital regions after DL. Increased posterior alpha activity is obtained in DL compared to repetitive training and baseline rest.

Figure 2 shows the mean power spectra for the theta, alpha, beta and gamma band. The ANOVA of theta responses revealed significant differences for training, *F*_(2,46)_ = 4.355, *p* = 0.019, $\eta_{p}^{2}$ = 0.16. *Post hoc* comparisons showed that the spontaneous EEG theta power was significantly higher in DL compared to repetitive training, *p* = 0.001 and baseline rest, *p* = 0.02. The ANOVA of theta responses revealed significant differences between locations, *F*_(4,92)_ = 3.134, *p* = 0.018, $\eta_{p}^{2}$ = 0.12. *Post hoc* comparisons showed that the spontaneous EEG theta power at frontal, central, parietal and occipital electrodes was higher than that of temporal electrodes, *p* = 0.05. The ANOVA of theta responses revealed significant results for training × location, *F*_(4,92)_ = 3.390, *p* = 0.012, $\eta_{p}^{2}$ = 0.13. *Post hoc* comparisons showed that in DL theta power was significantly increased at central, *p* = 0.008, parietal, *p* = 0.017 and occipital electrodes, *p* = 0.024, compared to repetitive training, and at central, *p* = 0.009, parietal, *p* = 0.011 and occipital electrodes, *p* = 0.019, compared to baseline rest.

The ANOVA of alpha responses revealed highly significant differences for training, *F*_(2,46)_ = 4.655, *p* = 0.009, $\eta_{p}^{2}$ = 0.17. *Post hoc* comparisons showed that the spontaneous EEG alpha power was higher in DL than in repetitive training, *p* = 0.007 and compared to baseline, *p* = 0.01. The ANOVA of alpha responses revealed significant differences between locations, *F*_(4,92)_ = 2.311, *p* = 0.014, $\eta_{p}^{2}$ = 0.13. *Post hoc* comparisons showed that spontaneous EEG alpha power was higher at central, *p* = 0.01, temporal, *p* = 0.01, parietal, *p* = 0.008 and occipital electrodes, *p* = 0.01, than that of frontal electrodes. A significant difference was obtained between parietal and frontal, *p* = 0.01, central, *p* = 0.03, temporal, *p* = 0.04 and occipital electrodes, *p* = 0.04. The ANOVA of alpha responses revealed significant results for training × location, *F*_(4,92)_ = 2.871, *p* = 0.027, $\eta_{p}^{2}$ = 0.11. *Post hoc* comparisons showed that in DL alpha power was significantly increased at central, *p* = 0.01, parietal, *p* = 0.02 and occipital electrodes, *p* = 0.04, compared to repetitive training.

The ANOVA of beta responses revealed significant differences for training, *F*_(2,46)_ = 3.653, *p* = 0.034, $\eta_{p}^{2}$ = 0.14. *Post hoc* comparisons showed that the spontaneous EEG gamma power was higher in the DL condition, compared to repetitive training, *p* = 0.04 and baseline rest, *p* = 0.03. The ANOVA of gamma responses revealed significant differences between locations, *F*_(4,92)_ = 2.676, *p* = 0.037, $\eta_{p}^{2}$ = 0.11. *Post hoc* comparisons showed that the spontaneous EEG beta power at temporal and occipital electrodes was higher than that of frontal, *p* < 0.05 each, central, *p* = 0.05 each and temporal electrodes, *p* < 0.05 each. No difference was obtained between temporal and occipital electrodes. The ANOVA of beta responses revealed no significant results for training × location.

The ANOVA of gamma responses revealed significant differences for training, *F*_(2,46)_ = 3.298, *p* = 0.041, $\eta_{p}^{2}$ = 0.13. *Post hoc* comparisons showed that the spontaneous EEG gamma power was higher in the DL condition, compared to repetitive training, *p* = 0.04 and baseline rest, *p* = 0.04. The ANOVA of gamma responses revealed significant differences between locations, *F*_(4,92)_ = 3.003, *p* = 0.022, $\eta_{p}^{2}$ = 0.12. *Post hoc* comparisons showed that the spontaneous EEG gamma power at temporal, and occipital electrodes was higher than that of frontal, *p* < 0.05 each, central, *p* < 0.05 each and temporal electrodes, *p* < 0.05 each. No difference was obtained between temporal and occipital electrodes. The ANOVA of gamma responses revealed no significant results for training × location.

## Discussion

The literature includes several previous investigations on the beneficial effects of DL on movement performance over repetitive training. To our knowledge, this is the first study that investigated post-training effects in DL on EEG brain activity. The present study was the first study that investigated acute post-training local EEG changes in DL and repetitive training. Our results clearly demonstrated different patterns of post-training EEG brain activity in DL and repetitive training. In DL, increases in frontal theta activity and occipito-parietal and central alpha activity compared to repetitive training and resting baseline were obtained. Repetitive training did not differ statistically from resting baseline in theta and alpha activity. The results from our study are in line with previous studies on effects of DL on EEG brain activity (Henz et al., 2013, 2014, 2015). In the following sections, we discuss different lines of argumentation to explain the found patterns of EEG brain activity after DL and repetitive training.

### Increased Post-Training EEG Theta and Alpha Activity Reflect Increased Learning Processes After DL

The observed differences in brain activation patterns indicate different neurophysiological processes in the acute formation of motor memory in DL and repetitive training. Our results are in line with previous studies, indicating that changes in EEG brain activity after movement performance and sensorimotor training compared to pretest and no-task conditions occur in general. These post-task traces in EEG brain activity are task-specific and are characterized by frequency-specific activation patterns (Ghilardi et al., 2000; Krakauer et al., 2000; Huber et al., 2004; Hung et al., 2013). For instance, Moisello et al. (2013) showed increases in frontal and posterior regions with alpha increases in the spontaneous EEG following a sequence-learning task. These post-task changes may represent a trace of motor learning and a correlate for use-dependent plasticity.

One line of argumentation is that the obtained increase in theta activity after DL indicates processes of learning and memory consolidation. Cortical and cortico-hippocampal theta phase synchronization found to characterize effective encoding have been postulated to facilitate simultaneous activation of neural assemblies (Fell and Axmacher, 2011). EEG theta rhythm has been reported to index prefrontal tagging of memories for subsequent consolidation during sleep (Benchenane et al., 2010). In recent studies on motor sequence learning, theta activity was beneficial on consolidation of motor learning (Reiner et al., 2014; Rozengurt et al., 2016). Accordingly, posttraining theta rhythm modulation might be a method of promoting procedural consolidation. Increases in theta and alpha activity in DL, compared to repetitive training indicate that the acute formation of motor memory is mediated by different neurophysiological pathways in DL and repetitive training. Extensive stimulation of the motor, and somatosensory areas in DL might increase post-training EEG theta and alpha traces. Further, the obtained pattern of low-frequency theta and alpha activation in central and posterior regions indicates that frontal cortical activity is down regulated in DL. In a recent study, it was shown that increased somatosensation reduced frontal cortical activity (Clark et al., 2014). A further explanation for a reduction of executively controlled processing in DL comes from the notion of the transient hypofrontality hypothesis (Dietrich, 2006). Its assumptions are that an extensive stimulation of the motor, somatosensory and visual areas prefrontal activity reduces cognitively controlled processing. In repetitive practice these processes might be stimulated to a lesser degree than in DL. It is well known that repetitive movement performance evokes habituational processes in the brain. In a recent study, it was shown that, after 10 min of uninterrupted finger movements paced by a metronome at 2 Hz, motor cortical excitability decreased. This decrease was induced by use (i.e., a sign of use-dependent plasticity), as this occurred without signs of neuromuscular fatigue (Crupi et al., 2013).

Under the assumption that motor learning leaves a specific local trace in the resting state EEG, we postulate that alpha increases might represent a first step towards long-term potentiation processes to consolidate memory. Further, we argue that in repetitive training learning, processes are stimulated less than in DL. One line of argumentation might be that due to repetitive movement performance habituation processes of the cognitive and motor system are to be postulated.

### Increased Theta and Alpha Activity Indicate Multisensory Processing in DL

Increased theta activity after DL might reflect multi-sensory processing (see Kanayama et al., 2015) due to high affordances on integration from information of different sensory modalities (visual, kinesthetic, proprioceptive) in DL compared with repetitive training. Direct communication of unisensory areas is supported by EEG-studies showing increased coherence between unisensory cortex areas during crossmodal processing. Hummel and Gerloff (2005) showed that synchronization between specific brain regions, as measured with EEG-coherence, is functionally significant for successful crossmodal integration. The idea of long-range synchronization during crossmodal processing is further supported by tasks requiring visuo-motor coordination. Comparing a visuo-motor tracking-task with either a motor-task combined with a visual distractor, a solely visual task or a sole motor-task without visual input revealed increased EEG-coherence between the visual and somatosensory or motor cortex areas during the visuo-motor tracking task compared to the other three conditions (Classen et al., 1998). From this point of view, we argue that DL strongly reinforces the construction of a visuo-somatosensory movement representation due to its affordances on processing of information from different sensory modalities. In contrast, in repetitive training, multisensory integration is fostered statistically less due to the large number of repetitions with the same movement configuration. A multisensory movement representation in DL might be a suitable explanation for better movement performance and stability of the movement representation against interferences from internal sources or external stimuli from the environment.

### Increased Theta Activity Indicates Enhanced Working Memory Processes in DL

Frontal-midline theta oscillations as measured by EEG recordings have been suggested as neural working language of executive functioning. Their power has been shown to increase when cognitive processing or task performance is enhanced. Enhanced cognitive processing is accompanied with increases of frontal-midline theta activity, specifically in tasks involving working memory (Mitchell et al., 2008) and executive functions (Nigbur et al., 2011). In addition, frontal-midline theta activity has been related to efficient working memory maintenance (Tóth et al., 2014). Further, increases of frontal-midline theta activity during task processing have been shown to enhance executive functioning (Sederberg et al., 2003) and reaction times in a Simon task involving conflict monitoring (Cohen and Donner, 2013).

The theta range has been described as a rhythm closely related to working memory processes. A recent study has shown that the oscillatory brain state predicts variability in working memory processes (Myers et al., 2014). Most of the research has been performed with experimental tasks, in which theta and alpha event-related synchronization (ERS) has been proposed as a possible carrier frequency for working memory processes (Sauseng et al., 2010). Theta rhythm is mainly seen in children. It decreases progressively with age, and it is enhanced while performing tasks involving attention and working memory (Carretié, 2001). Alpha activity has been shown to indicate processes of somatosensory and visual working memory (Haegens et al., 2011). Increased stimulation of sensory information processing would be a suitable explanation for the superior learning outcomes in differential compared to repetition-based training.

### Increased Theta Activity Indicates Enhanced Attentional Processes in DL

Finally, our results indicate that repetitive training and DL foster different attentional modes. Travis and Shear (2010) classify attentional processes reflected in the EEG in low- and high-frequency bands. Theta activity indicates open monitoring, whereas beta and gamma activity are related to focused attention (Wróbel, 2000; Basile et al., 2010). Therefore, increased theta activity in DL might reflect open monitoring. In a recent study it was shown, that relaxation techniques, i.e., audio-visual relaxation and autogenic training significantly improved athlete’s ability to perform a prolonged mental effort. These changes were accompanied by greater amplitude of waves in alpha band in the state of relaxation (Mikicin and Kowalczyk, 2015). From this, the obtained increase in frontal theta activity possibly reflects a brain state that is related to an attentional mode beneficial for motor learning and performance stimulated by DL.

A matter of discussion in recent research on sports performance is the role of particular brain states that lead to optimized motor performance (e.g., Dekker et al., 2014). For instance, Cooke (2013) showed that a particular brain state measured by patterns of brain activation is necessary for maximum motor performance. We argue that DL makes stable against disturbances stemming from internal or external sources. In a behavioral study, it was shown that the DL schedule prevents from C*hoking under pressure* as demonstrated in basketball free-throw (Lattwein et al., 2014). Two groups (DL, repetitive training) underwent a 4-week basketball free-throw intervention with three measurement points (pre-test, post-test, retention-test). At retention test, a simulation of a competitive setting (pressure condition) and a control condition (no pressure) was designed. The DL group significantly outperformed the repetitive training group in the pressure condition. Summarizing, we conclude that DL enhances attentional processes towards an optimum state of mind for motor learning and motor performance.

Further studies will address these points in depth and will elucidate the specificity, the time course and the long-term effects of DL on motor learning and performance and cognitive processes and the underlying neurophysiological processes.

## Author Contributions

The authors, DH and WIS cooperated on developing the theoretical framework and preparing the manuscript.

## Conflict of Interest Statement

The authors declare that the research was conducted in the absence of any commercial or financial relationships that could be construed as a potential conflict of interest.
